# Mineral Composition of Dietary Supplements-Analytical and Chemometric Approach

**DOI:** 10.3390/nu14010106

**Published:** 2021-12-27

**Authors:** Joanna Brzezińska-Rojek, Małgorzata Rutkowska, Justyna Brzezicha, Piotr Konieczka, Magdalena Prokopowicz, Małgorzata Grembecka

**Affiliations:** 1 Department of Bromatology, Faculty of Pharmacy, Medical University of Gdańsk, Gen. J. Hallera Avenue 107, 80-416 Gdansk, Poland; joanna.brzezinska@gumed.edu.pl (J.B.-R.); justyna.brzezicha@gumed.edu.pl (J.B.); 2 Department of Analytical Chemistry, Faculty of Chemistry, Gdańsk University of Technology, Gabriela Narutowicza 11/12 Street, 80-233 Gdansk, Poland; malgorzata.rutkowska@pg.edu.pl (M.R.); piotr.konieczka@pg.edu.pl (P.K.); 3 Department of Physical Chemistry, Faculty of Pharmacy, Medical University of Gdańsk, Gen. J. Hallera Avenue 107, 80-416 Gdansk, Poland; magdalena.prokopowicz@gumed.edu.pl

**Keywords:** beetroot, dietary supplements, mineral composition, toxic elements, health risk

## Abstract

There is a lack of data on the actual composition and effectiveness of beetroot-based dietary supplements. The research aimed to determine the profile of 22 elements (Na, K, Ca, Mg, P, Fe, As, Se, Zn, Cu, Ag, Co, Ni, Mo, Al, Mn, Sr, Cr, Ba, Li, Pb, Cd) in beetroot and its supplements by the microwave plasma atomic emission spectrometry (MP-AES) method. The analytical procedure was optimised and validated. The composition of both groups was compared, assessing compliance with the recommended daily doses for the chosen elements, and the health risk was estimated. Furthermore, chemometric analysis was applied. Beetroots constituted a significant source of elements, especially K, Na, Mg, Ca, P, in contrast to supplements which contained their negligible amounts except from iron-enriched products which provided notable amounts of Fe (38.3–88% of the Recommended Dietary Allowance for an adult male from 19 to 75 years old). Some products were significantly contaminated with toxic elements (As, Cd). Factor and cluster analyses were helpful in the differentiation of beetroot and its supplements in view of their type (vegetable, supplement, iron-enriched supplement), origin, type of cultivation (conventional, organic), and form (capsule, tablet) based on their mineral composition. The obtained results indicate the need for more stringent control of supplements, as they may pose a significant health risk to consumers.

## 1. Introduction

Beetroot (*Beta vulgaris* L.) belongs to the *Chenopodiaceae* family, consisting of approximately 1400 species, and within the genus, *Beta* L. abundant species are identified [1]. *Beta vulgaris* L. and its subspecies, such as the commercially available *B. vulgaris* ssp. *vulgaris*, *B. vulgaris* ssp. *maritima* and *B. vulgaris* ssp. *adanensis* are the most widespread. However, *B. vulgaris* ssp. *vulgaris*, known as beetroot, which is used both for industrial and non-commercial purposes, are the most relevant [2]. It is a biennial (rarely perennial) plant that produces an edible tuber with a colour that varies depending on the variety, from yellow to dark red [3], young leaves (chard), and seeds [2].

Beetroot is a rich source of nutrients (especially carbohydrates and proteins) with a relatively low energy value (43 kcal/100 g of fresh product) and bioactive ingredients; such as, betalains, inorganic nitrates, betaine, polyphenols, folates and elements [3]. Beetroot is especially rich in sodium (Na), potassium (K), calcium (Ca), magnesium (Mg), and phosphorus (P). Typically, the particular macromineral’s content is increasing in the following order P < Mg < Ca < K < Na. In addition, it is a good source of microminerals such as iron (Fe), zinc (Zn), manganese (Mn), and copper (Cu) [2,4,5].

Micro- and macrominerals are known to play important metabolic and physiological roles in the human body [6]. Sodium and K are essential to maintain the osmotic balance of body fluids, the body’s pH, regulate muscle and general nerve function, and control glucose absorption. Moreover, due to the good solubility of Na salts, it plays a crucial role in the transport of metabolites. Potassium is an antagonist of Na, and it exhibits a diuretic effect to maintain normal blood pressure by lowering it [4]. Calcium and P are significant for bone, tooth, and muscle growth and homeostasis. Moreover, Ca is an essential component of human blood and extracellular fluid; it is necessary for the proper functioning of the heart muscle, blood clotting, and neuromuscular transmission. Magnesium activates many enzyme systems and maintains the electrical potential in the nerves. It is essential in plasma and extracellular fluid, where it supports the osmotic balance [7]. Both Cu and Zn are components of many enzyme systems and participate in the formation of the immune response [8,9,10]. In addition, Cu is involved in Fe absorption, the proper functioning of the skeletal system, connective tissue, and blood vessels [8]. Zinc participates in the regulation of the cardiovascular system and the wound healing process [10]. Manganese is an activator of many enzymes. It participates in the functioning of the nervous system, blood clotting processes, cholesterol digestion, and it is a component of the skeleton [11]. *Beta vulgaris* L. also contains Fe, which is a component of many enzymes, but it is also part of the haem that forms the prosthetic groups of haemoglobin [12]. It takes part in the synthesis of hormones (such as serotonin, prostaglandins, thyroxine, and triiodothyronine), affects cholesterol metabolism, and promotes detoxification [13]. It also participates in the synthesis of DNA and plays a significant role in the immune system’s functioning [14,15].

Dietary supplements are becoming increasingly popular among consumers due to their ease of use, concentrated formula, and high availability on the market. The e-commerce sector has a significant share in the sale of dietary supplements [16]. In Poland, the registration procedure is relatively easy and free of charge, which additionally accelerates the development of the market [17,18]. For example, since 2020 (1 January 2020–31 August 2021), there have been 139 new formulations containing beetroot preserves reported to the Register of products subjected to the notification of the first marketing [19]. Mainly, they were only available via online sale, often through a single entity. The most common formulations were tablets, capsules, and powders. Despite the facile availability of fresh beetroot in Poland, there are many dietary supplements based on its preserves on the market. Therefore, it can be assumed that consumers frequently choose a condensed product over a vegetable. However, these products are not the same and consumers should be aware of this.

Since beetroot is a root vegetable, there is a risk of toxic elements accumulating in its products. Metals contamination might occur as a result of a single factor or a combination of different sources; such as, the characteristics of a plant and its growing conditions, the chemical composition of soil but also other features associated with conditions of harvesting, processing, manufacturing, storage, and transport [20]. Consumers may be particularly exposed to high levels of cadmium (Cd), mercury (Hg), and lead (Pb), which are considered toxic metals [21,22]. To protect consumers, WHO introduced Provisional Tolerable Monthly Intake (PTMI) for Cd [23]. Due to the high toxicity of As, its PTMI value was found to be no longer health-protective so benchmark dose (BMD) and the benchmark dose (lower confidence limit) (BMDL) are applied.

The study aimed to evaluate analytically and chemometrically the mineral composition of seventeen beetroot-based dietary supplements in comparison with fourteen beetroot samples. The determination of 22 elements (Na, K, Ca, Mg, P, Fe, As, Se, Zn, Cu, Ag, Co, Ni, Mo, Al, Mn, Sr, Cr, Ba, Li, Pb, Cd) in samples of beetroot (conventionally grown and organic) and beetroot-based supplements was conducted. The mineral composition of supplements was compared with vegetables (conventionally grown and organic). The safety and possible health benefits or risks of dietary supplements and vegetables consumption were assessed concerning the Adequate Intake (AI), the Recommended Dietary Allowance (RDA), PTWI, PTMI, BMDL values and the content of toxic elements in the analysed products was assessed in view of the regulations of the European Commission regarding contaminants in foodstuffs [24,25]. Furthermore, multivariate techniques were adopted in the differentiation of beetroot and its supplements in view of their type, origin, type of cultivation, and form. The Spearman’s rank correlation analysis and the Kruskal–Wallis test were applied to find correlations between contents of the analysed elements in samples. Then, chemometric techniques such as factor analysis (FA) and cluster analysis (CA) were used to classify beetroot and beetroot-based dietary supplements samples according to their type, origin, type of cultivation, and form.

## 2. Materials and Methods

### 2.1. Sample Preparation

Seven beetroots product portions were purchased in small-retail stores, large-retail stores (sales area > 400 m^2^), or grocery stores in Gdańsk (Poland, Europe) from November to December 2019. Three of them were marked as organic products and four were the result of conventional cultivation. Vegetables were washed, then peeled and chopped with ceramic tools (to avoid contamination with metal compounds, especially iron). Three samples were prepared from every batch. A total of twenty-one vegetable samples were analysed. All vegetables portions were frozen (−30 °C) and then lyophilized (Alpha 1–4 LD plus freeze dryer; −42 °C, 0.1 mbar, 170 h and 20 min of drying off in −50 °C, 0.02 mbar). Next, the samples were homogenised in porcelain mortars directly before analysis. Full characteristics of collected beetroot samples was presented in supplementary material in Table 1.

Seventeen commercially available supplements (from nine different manufacturers) made of beetroot or beetroot preserves were obtained from various drugstores or online stores from the Polish market. The complete characteristics of the analysed supplements was shown in Table 2. Products from 1GyA to 9SoB were capsules and from 6HeA to 8Sw were tablets. The letters A and B represent different serial numbers of the same product (or when it was not possible–from two different selling sources: 1GyA and 1GyB). The six products were enriched in iron compounds in which their content has been specified on the label (marked with a star in Table 2). Only products that met the criteria such as availability in the form of capsules or tablets, the presence of beetroot preserve (i.e., dried juice, powdered root, dried extracts, lyophilizate) as a main ingredient, availability for the Polish consumer via Internet sale or stationary in a drugstore were chosen for analysis. The assembled group was a representative group of the beetroot dietary supplements market in Poland, provided that selected criteria were taken into account. Every product was analysed in triplicate; thus, fifty-one samples of supplements were determined.

To determine the content of selected elements in the samples, they were subjected to microwave-assisted mineralisation (Anton Paar Multiwave Go microwave mineraliser, Anton Paar, Graz, Austria). For this purpose, about 0.5 g of the sample was weighed into a reaction vessel. Then 8 mL HNO_3_ was added to each reaction vessel. Mineralisation proceeded for the first 20 min at 100 °C and the next 20 min at 180 °C. Then the mineralised samples were placed in 25 mL flasks and replenished with deionised water (Millipore–Milli-Q Water Purification System, Merck, Darmstadt, Germany,) to the dash. After mixing, each solution was poured into stoppered plastic tubes.

A total of twenty-one vegetable samples and fifty-one dietary supplements were analysed.

### 2.2. Reagents and Standards

Potassium, Ba, Ca, Cd, Co, Cu, Zn, Ni, Pb, Se, and Mo standards at a concentration 1000 ± 2 mg/L, Mg standard solution at a concentration 1006 ± 4 mg/L, P standard at a concentration 1001 ± 3 mg/L, Fe standard at a concentration 1001 ± 2 mg/L, and Al standard at a concentration 998 ± 5 mg/L were obtained from Sigma Aldrich (Merck, Darmstadt, Germany). Na and Li standards at a concentration 10,000 mg/L, Sr standard at a concentration 1005 ± 5 mg/L in 4% HNO_3_ were purchased from Ms Spectrum (Warsaw, Poland). Ag standard at a concentration 1000 ± 5 mg/L was purchased from Fluka Analytical (Merck, Darmstadt, Germany). As standard at a concentration 1000 mg/L in 2% HNO_3_ was obtained from Thermo Fisher Scientific Inc. (Göteborg, Sweden). Cr standard at a concentration 1003 ± 3 μg/mL and Mn standard at a concentration 1000 ± 6 μg/mL were purchased from CPI INTERNATIONAL (Santa Rosa, CA, USA). Nitric acid (65–70% purity) was obtained from Alfa Aestar (Merck, Darmstadt, Germany). Certified reference materials: M–4 CormTis, M–3 HerTis and M–5 CodTis were supplied by LGC Standards. DOLT-4 was purchased from the National Research Council of Canada (NRC-CNRC) (Ottawa, ON, Canada).

### 2.3. Determination Procedure

The determination of the elements in the tested samples was carried out using atomic emission spectrometry with microwave plasma atomisation (the 4210 MP-AES supplied by Agilent) at specific wavelengths for each element (Table 3). Determinations were made at several wavelengths for each element. The final choice of the wavelength at which the determination was made was determined by the value of the coefficient R^2^ for the calibration curve. 

### 2.4. Method Validation

The MP-AES method was validated by linearity range, precision, accuracy, the limit of determination (LOD), and the limit of quantification (LOQ). The LOD and LOQ of the applied method were calculated using formulas proposed by Huber [26]:

(1) $$\mathrm{LOD}=\frac{3.3{\;\mathrm{SD}}_{a}}{b}$$

SD_a_—standard deviation of the intercept for the calibration curve;
b—slope for the calibration curve;

When calculating the numerical limit of quantification (LOQ), the dependence described by equation [26] was used:

(2) LOQ=3·LOD

The validation parameters were presented in Table 3. The determination coefficients (R^2^) were in the range of 0.9857–0.9999. Accuracy was determined based on CRMs (M–4 CormTis, M–3 HerTis, M–5 CodTis, DOLT-4) analysis and was expressed as recovery. The average recovery for the selected elements (Na, K, Ca, Mg, P, Fe, As, Se, Zn, Cu, Ag, Co, Ni, Mo, Mn, Sr, Cr, Ba, Li, Pb, Cd) was in the range of 80–120% and we can regard these as acceptable values for this type of analysis (Table S1). The precision was calculated as the coefficient of variation for all the results obtained in all the analysed samples. Values were obtained at an acceptable level and did not exceed 10%. Recovery for calibration curves (R_cc_) was calculated based on signal obtained for standards (S_expected_) and signal calculated from calibration equation (S_calculated_) according to the formula:

(3) $$R_{\mathrm{cc}}=\frac{\left|S_{\mathrm{expected}}-{\;S}_{\mathrm{calculated}}\right|}{S_{\mathrm{expected}}}$$

### 2.5. Calculations

#### 2.5.1. Content Calculations

The content of individual elements was determined in µg/g of dry weight (for beetroot samples) and µg/g of product for supplements. Then, the content of individual elements in beetroot samples was recalculated to µg/100 g of fresh weight (f.w.) using the water content values (Table 1). The detailed results of conducted analyses are presented in Tables S2 and S3. In the case of the supplements, the content of the analysed elements was expressed in µg per dosage unit (d.u.) (Table S3). Values in Tables S2 and S3 were expressed as the mean content in a product ± expanded uncertainty (U) of measurement at a 95% confidence level obtained for three replicates.

#### 2.5.2. Intake Assessment

The consumption of the selected elements with the analysed products was evaluated based on the estimated daily intake (EDI). It was assumed that 100 g of beetroot was consumed daily (EDI). For supplements, EDI was calculated based on the recommended intake declared by the manufacturers (Table 2). The estimated weekly intake (EWI) was calculated by multiplying the EDI by 7 which equates to 7 days, while the estimated monthly intake (EMI) by multiplying the EDI by 30.

#### 2.5.3. The Realisation of Dietary Recommendations and Health Risk Assessment

The realisation of the daily nutritional recommendations was assessed with the consumption of the analysed products based on the Adequate Intake (AI) or Recommended Dietary Allowance (RDA) value for an adult male (from 19 to 75 years old) according to recommendations for the Polish population [27]. For vegetables, a portion of 100 g fresh vegetables was applied. For supplements, the calculation was based on the manufacturer’s recommended daily intake. Moreover, in the case of iron-enriched supplements (six products), the compliance of the iron content with the manufacturers’ declarations was calculated and expressed as a percentage. Then, values were compared with the guidelines implemented by the European Commission in 2012 on establishing tolerance limits for minerals contained on labels [28,29].

The content of selected toxic metals in products was assessed in view of the regulations of the European Commission No 1881/2006 and No 629/2008 [24,25]. In addition, human exposure was evaluated by relating the EDI index to the PTWI, PTMI, or BMDL values. The United States Pharmacopoeia (USP 43-NF 38) recommends manufacturers of supplements to assess the content of elemental contaminants (As, Cd, Hg, Pb) and estimate the health risk based on Provisional Tolerable Weekly Intake (PTWI) that is recommended by the Food and Agriculture Organization of the United Nations and World Health Organization (FAO/WHO) [30].

#### 2.5.4. Statistical Analysis

The methods of statistical analysis were chosen after verification of the normal distribution using the Shapiro–Wilk test [31]. The obtained data were not normally distributed and, therefore, non-parametric tests, i.e., the Spearman’s rank correlation analysis, and the Kruskal–Wallis test were applied. Statistically significant results of the Kruskal–Wallis test showed that at least one group is different from another group. To verify the results of the Kruskal–Wallis test, a post-hoc Dunn’s test was performed on the obtained database. The post-hoc test was used to pinpoint which specific means were significant from the others. There were also performed, factor analysis (FA) along with the cluster analysis (CA). They were used to identify the main components underlying groups’ differences. All analyses were done using Statistica 13.3 (TIBCO Software Inc., Palo Alto, CA, USA). The analysed data were standardized [32,33] and arranged in columns (elements) and rows (the analysed beetroot samples and dietary supplements). The obtained database allowed to perform a series of factor analyses of all tested samples (beetroot and dietary supplements), all elements (descriptors) were used. Cluster analysis was performed for all samples (beetroot and dietary supplements) and dietary supplements alone. Ward’s method and Euclidean distance were used in the CA.

## 3. Results and Discussion

The research aimed to determine the profile of 22 elements (Na, K, Ca, Mg, P, Fe, As, Se, Zn, Cu, Ag, Co, Ni, Mo, Al, Mn, Sr, Cr, Ba, Li, Pb, Cd) in twenty-one beetroot samples and fifty-one beetroot-based dietary supplements samples using the MP-AES method. Then the mineral composition of supplements was compared with beetroot samples. The safety, possible health benefits, or risks of dietary supplements and vegetables consumption were assessed in view of Polish and European regulations.

### 3.1. Content of the Analysed Elements

The average results for two groups of beetroot samples (conventional and organic cultivations) are presented in Table 4. The mean results for dietary supplements in both forms, i.e., capsules and tablets, are shown in Table 5. The minimum and maximum concentration of the determined elements, the mean content with the standard deviation for the group, and the number of samples with the content of the analysed element above the LOQ are given. Results were expressed as mg/100 g fresh weight for beetroot samples and as µg/dosage unit for dietary supplements (content was determined in µg/g of product and then recalculated into dosage unit). The concentration of Ag, Co, Cr, Li, Mo, Ni, Pb in conventional and organic beetroots samples were determined under the LOQ (Table 4). The observed variability in the analysed mineral concentration might be a result of the differences in plants’ geographical origin [34], fertilization [35,36], as well as varietal differences [37]. In the case of dietary supplements, Se, Cu, Ag, Co, Cr, Ba, Li, Mo, Ni, Pb concentrations were under the LOQ (Table 5). Moreover, none of the tablet products contained Zn and As above the LOQ. The variability in individual groups might be related to the different origins of the supplements, the content of beetroot products, and beetroot preserves used in production. Products, mainly of Polish origin (one product manufactured in the USA), were purchased from different online pharmacies. In addition, the products sourced from various manufacturers contained different auxiliary substances (such as anti-caking agents, acidity regulators, and sweeteners), and the dosage unit size varied. There are no literature data that would allow the comparison of the mineral composition of beetroot-based dietary supplements, therefore, beetroot samples were used for this purpose.

#### 3.1.1. Macrominerals in Beetroots and Dietary Supplements

Potassium was the most abundant macromineral in beetroot and beetroot dietary supplements, i.e., 266 mg/100 g and 3.51 mg/d.u., respectively (Table 4; Table 5). In turn, the content of P, Mg, and Ca in beetroot samples ranged from 20.8 to 22.4 mg/100 g. In the case of dietary supplements, the content of these elements was more diversified (3.30–8.71 mg/d.u.). The conventional and organic beetroots showed comparable contents of Na (35 mg/100 g and 32 mg/100 g) but lower than those obtained by Chhikara et al. (77 mg/100 g) [2]. However, the analysed organic samples were richer source of K (356 mg/100 g), P (38 mg/100 g), Mg (30 mg/100 g), Ca (34 mg/100 g) than conventional ones-K (266 mg/100 g), P (19 mg/100 g), Mg (22 mg/100 g), Ca (22 mg/100 g) (Table 4). Interestingly, the K content in organic samples (356 mg/100 g) was higher than the one reported by Chhikara et al. (305 mg/100 g) [2]. This difference may be related to the varied content of this element in the soil as well as the use of potassium fertilizers [35,38]. The highest concentration of Na, K, P, Mg, Ca was found in organic sample 2Bo (Table S2).

The content of macrominerals in the supplements differed between the products. The highest Na content among the capsules was determined in product 9SoA (6040 µg/d.u.) and 9 SoB (6947 µg/d.u.) samples, which contained 10–20 times more Na than the others (Table S3). Among the tablets, product 8SwA (3276 µg/d.u.) contained the highest amount of Na. All the analysed supplements, both in capsules and tablets, were characterised with a much lower macromineral content per dosage unit than the 100 g of fresh beetroot. Even considering that some supplements are recommended to be taken in several dosage units, the total daily intake of macrominerals is incomparably small with the portion of the analysed beetroots. For example, to provide a comparable amount of Na with a product 9SoB (Table S3) product as with a 100 g serving of an average beetroot (35 mg/100 g, Table 4), a consumer should take five capsules of this product (the manufacturer recommends 2 capsules/day; d.u. = 0.69 g). In addition, auxiliary substances used in the product formulation might be the source of some elements in supplements as manufacturers use sodium, magnesium, calcium, silicon compounds (Table 2). For example, the product 8SwA contained much more Ca (1771 µg/d.u.) than the other supplements from both groups and was the only one that contained calcium salts of fatty acids, marked as an anticaking agent on the label, in its composition (Table 2).

#### 3.1.2. Microminerals in Beetroots and Dietary Supplements

Both conventional and organic beetroots constituted a substantial source of Mn (0.58 mg/100 g and 0.36 mg/100 g) (Table 4). Similar results were found by Lisiewska et al. (0.39 mg/100 g) [39] and Ekholm et al. (0.40 mg/100 g) [40]. Moreover, conventional beetroots contained Se (0.54 mg/100 g), Zn (0.38 mg/100 g), and Cu 0.10 mg/100 g) which were under the LOQ in organic samples. Organic samples were a better source of Fe (0.88 mg/100 g) than conventional ones (0.70 mg/100 g) and the obtained values were comparable with those reported by Grembecka et al. (0.99 mg/100 g) [41].

Dietary supplements contained insignificant amounts of microminerals as compared to the analysed beetroots. Selenium, Cu, Ba concentrations were under the LOQ in these products (Table 5). Furthermore, tablets did not contain Zn above the LOQ (0.96 µg/g). Zinc was determined only in two capsules products–3GaA (3.88 µg/d.u.) and 6HeB (4.53 µg/d.u.). Dietary supplements contained from 9.5 to 13 µg/d.u. of Mn (Table 5), which was around 20 times less than in a portion of a raw vegetable (Table 4). The richest sources of Mn among supplements were product 3GaA (19.75 µg/d.u.) and 3GaB (19.38 µg/d.u.). At the same time, these two formulations contained on average 15–20 times higher amounts of Al (222 µg/g and 227 µg/g) (Table S3) than others. The elevated concentration of Al in the beetroot-based dietary supplements might be a result of contamination of the cultivation area where beetroots were grown before processing due to anthropogenic activities such as car exhaust fumes, dust from coal combustion, waste, and the steel industry [42,43,44]. Contamination could also occur at the stage of product processing or packaging [44]. Only iron-enriched dietary supplements (Table S3) characterised with much higher amounts of Fe (1.28–2.80 mg/d.u.) than raw vegetables (0.68–0.82 mg/100 g f.w.).

### 3.2. Realisation of Dietary Recommendations

To assess the nutritional and pro-health value of the analysed products, the percentage of the Recommended Dietary Allowance (RDA) or Adequate Intake (AI) was calculated for the selected elements. The calculations were made based on the nutritional recommendations for the Polish population [27] for an average male from 19 to 75 years old. There are no RDA guidelines for Na, K, Mn, so it was decided to use the AI values for these elements. It was assumed that a 100 g serving of beetroot or recommended amount of dietary supplement by the manufacturer was consumed.

#### 3.2.1. Realisation of Dietary Recommendations by Analysed Beetroots

Table S4 shows the detailed realisation of dietary recommendations by the analysed beetroot samples, while Table 4 presents a summary for the groups of conventional and organic cultivations. Realisation of AI (for Na and K) or RDA (for P, Mg, Ca) did not exceed 15% for all beetroot samples. The greatest realisation of recommendations for macrominerals was ensured by organic sample 2Bo (from 4.1% of AI for Na to 15% of RDA for K). Both, conventional and organic beetroots, were particularly rich sources of Mn and realized AI from 9.7% to 91% (Table S4). The sample 1Bo provided the highest amounts of Mn (91% AI for men). The sample 3Bo contained a significant amount of Se, which enabled the RDA to be reached at the level of 983%. Such a high Se content in this sample might be due to soil contamination reported for industrial grounds or careless fertilization with Se salts [45] as well as contamination during collection, packaging, or display in a store. The Tolerable Upper Intake Level (UL) of Se for adults (400 µg/day) [46] will also be exceeded (135%) assuming the consumption of 100 g of the sample 3Bo, which can be associated with the risk of selenosis with long-term exposure [47,48]. The realisation of RDA for Zn and Cu ranged from 3.2% to 11.5% and was ensured only by conventional samples (Table S4).

#### 3.2.2. Contribution to Mineral Intake by the Analysed Beetroot-Based Dietary Supplements

In general, the analysed beetroot-based dietary supplements constituted a minor source of micro- and macrominerals in comparison with a 100-g serving of any analysed beetroot. Table 5 shows the realisation of dietary recommendations by the analysed products: capsules and tablets. The realisation of AI for Na and K with a recommended portion of supplements did not exceed 1.5%. Similarly, capsules and tablets provided not more than 2.8% of RDA for P, Mg, and Ca. The realisation of RDA for Zn was lower than 0.12% and for Mn ranged from 1.1% to 4.9% of RDA for men.

**Figure 1:**
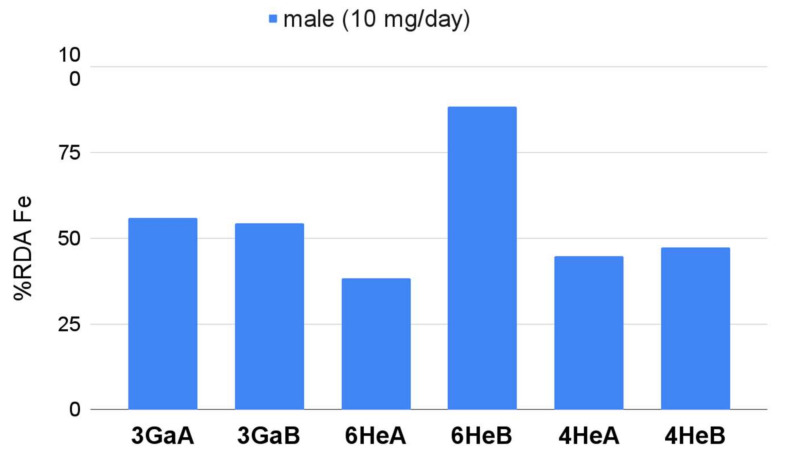
Realisation of dietary recommendations for Fe by iron-enriched beetroot-based dietary supplements. Recommendations for male (10 mg/day) were applied according to Jarosz et al. [27].

Only products enriched with iron compounds (3GaA, 3GaB, 6HeA, 6HeB, 4HeA, 4HeB) deserved attention, as they enabled the realisation of the RDA Fe in the range of 45–88% for men (Figure 1). The best iron source was the product 6HeB. All the analysed iron-enriched products contained organic salt of Fe-iron gluconate which is one of the most common Fe compounds used in dietary supplements [49]. Dietary iron comes in two forms: haem available in animal products (red meat, offal, liver) and non-haem present in plant-based products. The haem form is better absorbed by the body within the levels of 25–35% [50]. Its bioavailability is not affected by factors such as the content of Ca in the diet, phytates, or proteins, which reduce the absorption of non-haem iron to 2–20%. Dietary supplements might contain non-haem (Fe salts) and haem forms. The ferrous salts (sulphate, fumarate, and gluconate) are better absorbed (10–15% bioavailability) than ferric salts [50]. The bioavailability of Fe depends on the type of Fe salt, and that of iron gluconate amounts to 12% [51]. As a result, consumers should take approximately 150 mg of iron gluconate to provide RDA (18 mg/day [27]). Seiler et al. [52] reported that supplementation with 60–80 mg of Fe/day for 12 weeks may be an effective treatment of Fe deficiency in a healthy population. WHO recommended a dose of 30 to 60 mg of elemental Fe for menstruating, non-pregnant female adolescents, particularly in settings where the prevalence of anaemia is 40% or higher [53]. Taking into account the manufacturer’s recommended dosage, Fe-enriched supplements provided from 4.5 to 8.8 mg of elemental Fe (EDI), which means that they might not have a significant effect on the prevention of anaemia. By comparison, unenriched supplements provided no more than 4.1% of the RDA (Table S5) while the portion of the analysed beetroots–from 2.8% to 8.8% of RDA (Table S4).

### 3.3. Verification of Manufacturers’ Declarations on Fe Content

Producers declared that products 3GaA and 3GaB contained 2.8 mg Fe/d.u. while products 4HeA, 4HeB, 6HeA, 6HeB had 1.4 mg Fe/d.u. It was found that the compliance of iron content with the manufacturer’s declaration ranged from 91 to 210% (Table 6). The determined values of iron content in iron-enriched products were compared to the guidelines introduced by the European Commission in 2012 on establishing tolerance limits for minerals contained on labels amounting to −20 to +45% for a dietary supplement containing minerals [28,29]. Only one product (6HeB) did not meet the requirements.

There is little research into the compliance of the actual content of minerals in dietary supplements with producers’ declarations. Puścion-Jakubik et al. [54] investigated the content of Mg in dietary supplements with this mineral. They reported that the mineral concentration may vary significantly between information on the products’ labels and the determined values. The study evaluated that consumers may take up to 304% more Mg per day or 98% less than it was declared by the manufacturer. Similarly, Niedzielski et al. [55], who examined Se content in dietary supplements enriched in this micromineral, reported that Se concentration in 56% of the analysed products was not within the acceptable margin of the declared value To summarize, non-compliance of the minerals’ content with the manufacturers’ declarations may lead to side effects [48,56] as a consequence of excessive or insufficient consumption of minerals.

### 3.4. Health Risk Assessment

#### 3.4.1. Content of Toxic Elements in Samples vs. the European Commission Regulations

European Commission Regulations No 1881/2006 and No 629/2008 regulate the maximum levels of contaminants in foodstuffs, including Cd, Pb, and Hg [24,25]. The content of Cd and Pb in the analysed products was assessed in view of the above-mentioned regulations (Table 7). Lead contamination is allowed at the level of 0.10 mg/kg f.w. for vegetables and 3.0 mg/kg f.w. for the supplement. In the analysed samples, Pb was not determined above the LOQ (0.69 mg/kg). Cadmium contamination is allowed at the level of 0.06 mg/kg f.w. for vegetables and 1.0 mg/kg f.w. for the supplement. Cadmium was determined in 3 conventional beetroot samples, 3 capsules, and 2 tablet supplements. In all cases, the determined content greatly exceeded the permissible standards (Table 7). Detailed results for individual samples are summarized in Tables S2 and S3.

As a root vegetable, beetroot tends to accumulate Cd and Pb [57,58]. High Cd content in selected samples may indicate environmental pollution, where vegetables were grown [58]. Beetroot is considered one of the main sources of heavy metals in the diet, along with carrots [21,22,57]. Consumption of these products might be associated with a high health risk to the consumer. It can result in Cd accumulation in organs, especially in kidneys, leading to their failure. Moreover, Cd is classified as a human carcinogen (Group 1) [59].

#### 3.4.2. Health Risk Assessment for Population

Consumer’s exposure was assessed based on PTMI and BMDL indices for Cd and As, respectively. After the 2011 evaluation, the WHO withdrew the PTMI dose for As because it was no longer health protective. The health risk was estimated based on the BMDL_0.5_ value (3 µg/kg body weight), i.e., the dose that increased the incidence of lung cancer by 0.5% [23]. Due to equipment limitations, the speciation of As compounds was not evaluated. It was assumed that As was present in the samples mainly in the inorganic form [60], therefore, the BMDL values were referred to.

Arsenic was determined in three conventional beetroot samples (1Bo) and three organic beetroot samples (2Bo). The ratio of EDI to BMDL_0.5_ values (210 µg As/70 kg b.w.) assuming consumption of 100 g of beetroot amounted to 1546% of BMDL_0.5_ and 1573% of BMDL_0.5_ for conventional and organic samples, respectively. The values obtained indicate significant contamination of the material, which should not be consumed by consumers. Arsenic compounds can mainly come from contaminated soil and beetroot, as a root vegetable, tends to accumulate them [61].

**Figure 2:**
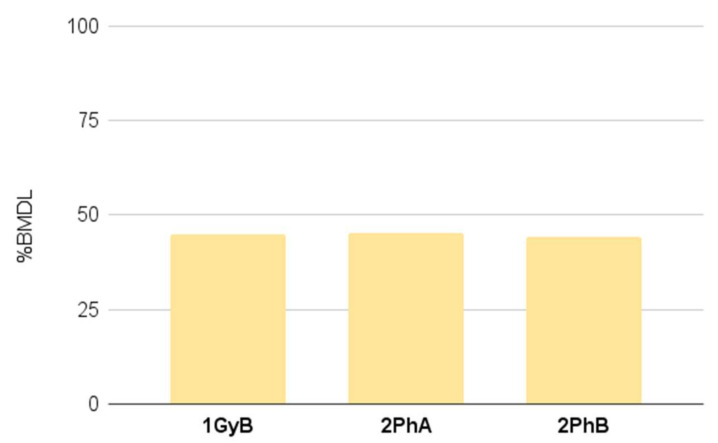
The ratio of EDI to BMDL value for the chosen dietary supplements (considering the recommended dosage/day by the manufacturer).

In the case of dietary supplements, As was determined in three capsulated products (1GyB, 2PhA, 2PhB), which were distributed by the same manufacturer. Figure 2 shows the ratio of EDI to BMDL_0.5_ value, assuming that the consumer will take the supplements according to the manufacturer recommendations (1 d.u./day, Table 2). Although the BMDL_0.5_ value was not exceeded, the obtained results (44–56% of BMDL_0.5_) indicate products’ contamination, and their consumption might be associated with a significant risk to the consumer.

Consumer’s exposure to Cd was assessed based on PTMI value (25 µg/kg b.w./month) [23]. This toxic metal was determined in three conventional beetroot samples (5Bo). The ratio of EMI to PTMI values (1750 µg Cd/70 kg/month), assuming monthly consumption of 100 g of beetroot by an average adult weighing 70 kg, constituted 109% of PTWI. This indicates significant contamination of the material that should not be consumed by consumers. Cadmium, which can originate mainly from contaminated soil, might be accumulated by beetroot, a root vegetable [62]. Moreover, Sekara et al. [58] showed that the accumulated cadmium is distributed between beetroot tissues in proportion to the content of Cd in the soil.

**Figure 3:**
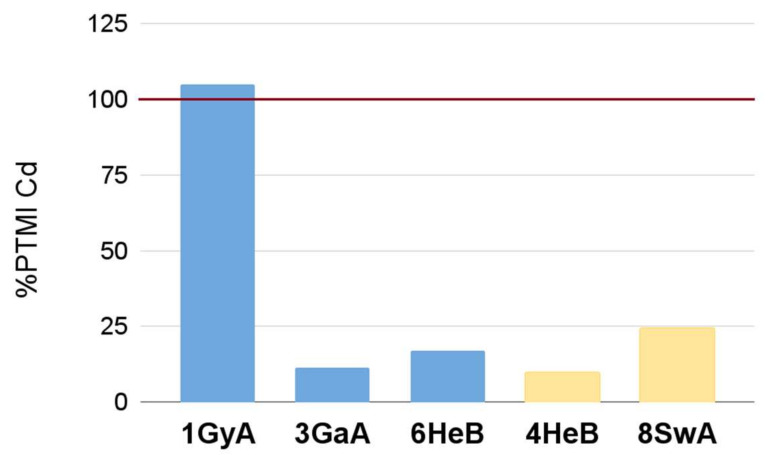
The ratio of EMI to PTMI values for the chosen dietary supplements (taking into account consumption of the recommended dosage/month by an average adult weighing 70 kg). The blue bars are for capsule supplements and the yellow bars are for tablets.

Cadmium was determined in three capsulated supplements (1GyA, 3GaA, 6HeB) samples and two in tablet form (4HeB, 8SwA). Figure 3 shows the ratio of EMI to PTMI values (1750 µg Cd/70 kg/month), assuming that an average adult weighing 70 kg will follow the producers’ recommendations concerning dosage for a month. Only one product (1GyA) exceeded the PTMI value for Cd. However, the other four supplements were also substantially contaminated with this metal and can constitute an important source of Cd in the diet. They should be included in the risk assessment of human exposure to Cd compounds.

### 3.5. Statistical Analysis

#### 3.5.1. Correlation Analysis

Correlation analysis was used to study the strength of a relationship between particular variables, i.e., macro- and microminerals. The non-parametric Spearman’s rank test was used at three significance levels, i.e., *p* < 0.05, *p* < 0.01 and *p* < 0.001 (Supplementary Materials Tables S6–S8). Negative and positive correlations were found between the analysed elements in all three datasets, i.e., all samples, beetroot samples and dietary supplements’ samples. The highest number of positive correlations (*p* < 0.001) was found in a database of all the analysed samples, i.e.,: Na-K, K-P, K-Ca, K-Mg, K-Mn, K-Sr, K-Ba, P-Ca, P-Mg, P-Mn, P-Sr, P-Ba, Fe-Mn, Ca-Mg, Ca-Mn, Ca-Sr, Ca-Ba, Mg-Mn, Mg-Sr, Mg-Ba, Mn-Sr, Mn-Ba and Sr-Ba. Beetroot samples were characterized with positive correlations (*p* < 0.001) between Fe-Ca, Fe-Mg, Fe-Al, Fe-Mn, Fe-Ba, Ca-Mg, Mg-Mn and Mg-Ba. Positive correlations between minerals indicates similar plant uptake rates when using different plant channels. In the case of dietary supplements, no similar correlations were found, despite the presence of the main ingredient originating from the analysed vegetable. Strong interdependences (*p* < 0.001) were found for Mg-K, Mn-Fe, and Sr-Ca in dietary supplement samples. The occurrence of these correlations might be influenced by the very different matrix of these products, thus, emphasizing the role of other components such as excipients.

#### 3.5.2. Kruskal–Wallis Test

The Kruskal–Wallis test allowed to show statistically significant differences in the analysed database. Different categories were subjected to this test. The first one concerned all the analysed samples in view of the product type (vegetable-supplement) taking into account the fact of iron-enrichment. The second category concerned beetroot, in which samples were classified according to their cultivation method (conventional and organic) and geographical origin. In the case of the third classification, the samples of dietary supplements were categorized according to the type of the main component (extract, root, and juice) and the presence of the enriched ingredient (iron). The results of the Kruskal–Wallis test for the samples (vegetable-beetroot and dietary supplements, including enriched and non-enriched) showed relationships at three levels of significance: *p* < 0.001, *p* < 0.01, and *p* < 0.05. The relationship between the form of the product (vegetable-beetroot and dietary supplement) and the elemental content of the analysed samples was found in case of Ba, Sr, Ca, K, Fe, Mn, Mg, P (*p* < 0.001) and Na (*p* < 0.01). The origin of beetroot was related to the content of Sr, Ba, K, Ca, Mg (*p* < 0.001), Mn, Fe (*p* < 0.01) and Na (*p* < 0.05) (Table S9). The type of main component of dietary supplement significantly influenced the content of Mn (*p* < 0.01), Fe, Mg and Sr (*p* < 0.05).

#### 3.5.3. Post-Hoc Dunn’s Test

A post-hoc test, i.e., Dunn’s test, was performed to pinpoint, which specific means are significant from the others. The results were presented in Table S10. This test was applied for all samples (beetroot and dietary supplements) and each dataset separately. In the case of beetroot samples, the Dunn test (*p* < 0.05) showed a significant relationship for Sr and samples from Greater Poland and Mazovia. Pharmaceutical form (capsules-tablets) of dietary supplements was associated with K, Mg, and Al. The presence of these elements might be related to the usage of auxiliary substances (such as anti-caking agents, acidity regulators, and sweeteners) or contamination during processing (Al). The dependence of the type of the supplement main component (enriched extract–non-enriched extract-root-enriched juice–non-enriched juice) was demonstrated for Mg (non-enriched juice-root).

#### 3.5.4. Factor Analysis

**Figure 4:**
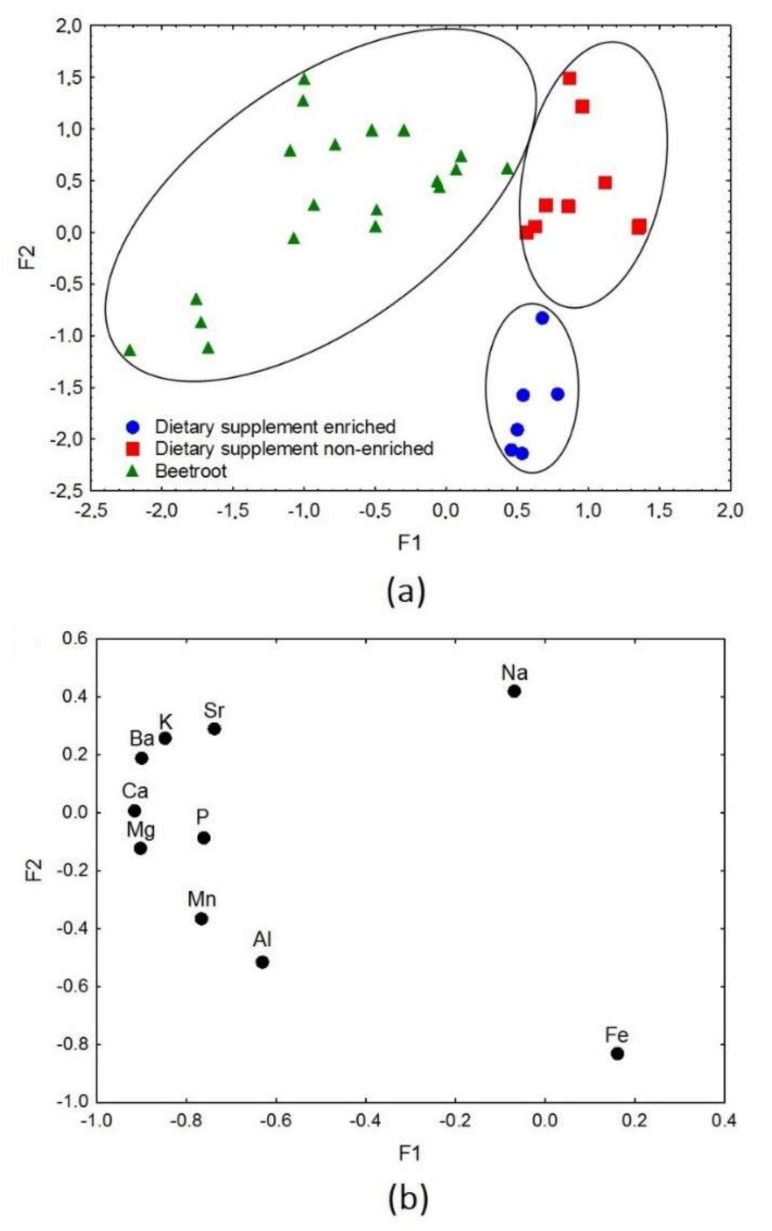
(**a**) Scatter plot of object samples of two factors for the dataset of beetroots and all dietary supplements. (**b**) Scatter plot of loading for elements in all the analysed samples.

First, factor analysis was performed for all the analysed samples containing beetroot samples and dietary supplements (enriched and non-enriched with iron), for which results were presented in Figure 4a,b. The analysis included all the analysed elements. The value of the first factor (F1) of the explained variance amounted to 53.2%, while the second factor (F2) amounted to 14.8%. Both factors cumulatively explained 68.0% of the total variance, whereas the eigenvalues for F1 and F2 were 5.32 and 1.48, respectively. As can be seen in Figure 4a, factor 1 (F1) distinguishes samples based on their category, i.e., beetroot samples from dietary supplements ones. Lower values of F1, described by Ba, K, Sr, Ca, Mg, P, Mn, and Al, corresponded to the beetroot samples (Figure 4b). The elements that characterized the group of dietary supplements (enriched and non-enriched with iron), described by higher F1 values, were Na and Fe. Factor 2 (F2) was responsible for the distribution of dietary supplement samples according to their iron-enrichment. Lower F2 values, described by Fe, characterized the enriched dietary supplements (Figure 4b). Na, which is attributed to higher F2 values, corresponds also to non-enriched supplements. It is probably related to the presence of Na in the auxiliary substances’ composition found in supplements. Based on the results, it can be concluded that the consumption of beetroot will not provide the same elements as dietary supplements, even though it is the main ingredient of the latter. Beetroots’ consumption in the form of unprocessed vegetables provides the body with greater amounts of elements (K, Ca, Mg, P, Mn, Al, Ba, and Sr), which also translates into the greater variety of these samples compared to dietary supplements (Na and Fe).

**Figure 5:**
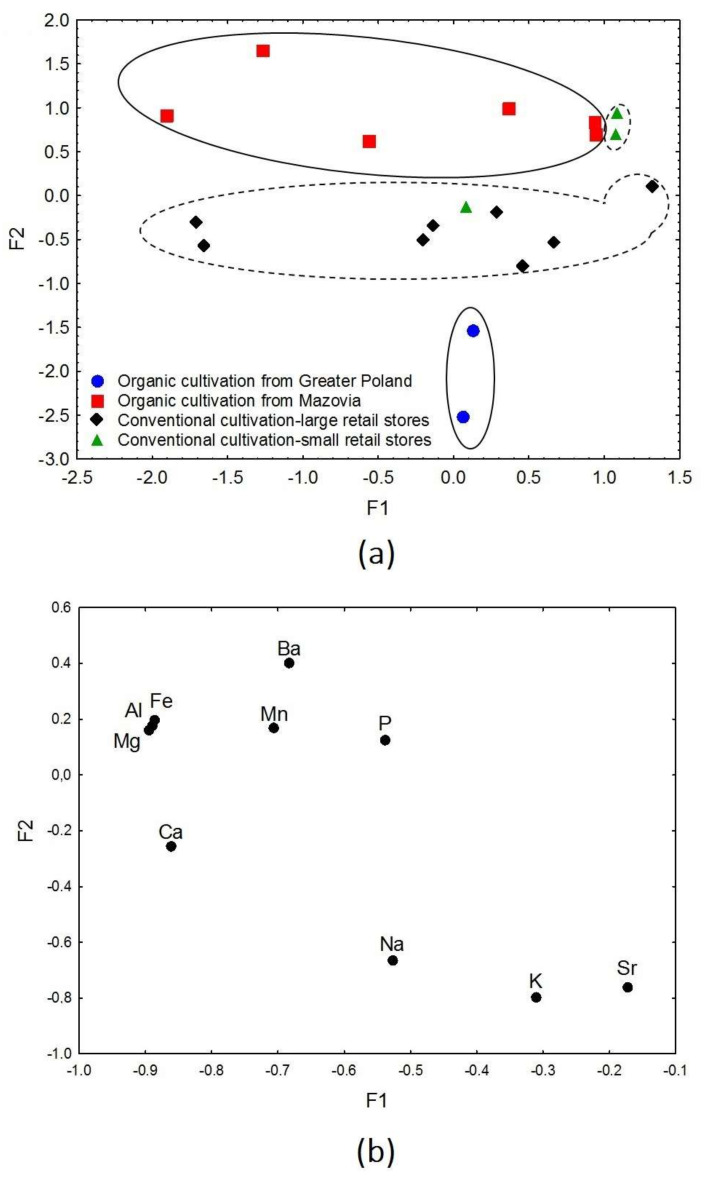
(**a**) Scatter plot of object samples of two factors for the beetroot samples. (**b**) Scatter plot of loading for elements in all the analysed samples.

Based on the results of the first factor analysis, further analyses were performed to obtain detailed information concerning datasets of beetroot samples and dietary supplements. Factor analysis of beetroot samples diversified samples into products grown conventionally and organic (Figure 5a,b). In addition, a clear separation of beetroot samples cultivated organically because of the geographical origin was also obtained. There were also distinguished samples grown conventionally from large and small-retail stores. It was found that 68.0% of the total variance was explained by F1 (47.8%) and F2 (20.2%). The eigenvalues were 4.78 and 2.02 for F1 and F2, respectively. Factor 2 was responsible for the diversification of the organic beetroot samples from different geographic origins (Mazovia-Greater Poland), as well as conventional samples from stores of various size scales (Figure 5a). Organic beetroot samples from the Mazovian area and conventional samples from small-retail stores were characterized by higher F2 values and Mg, Al, Fe, Mn, Ba, and P (Figure 5b). Lower F2 values (described by Na, K, and Sr) corresponded to the organic samples from Greater Poland and conventional ones purchased in large-retail stores. Due to the limited information about the product provided by the supplier, it was not possible to explain the influence of the factor F1 on the distribution of the samples.

**Figure 6:**
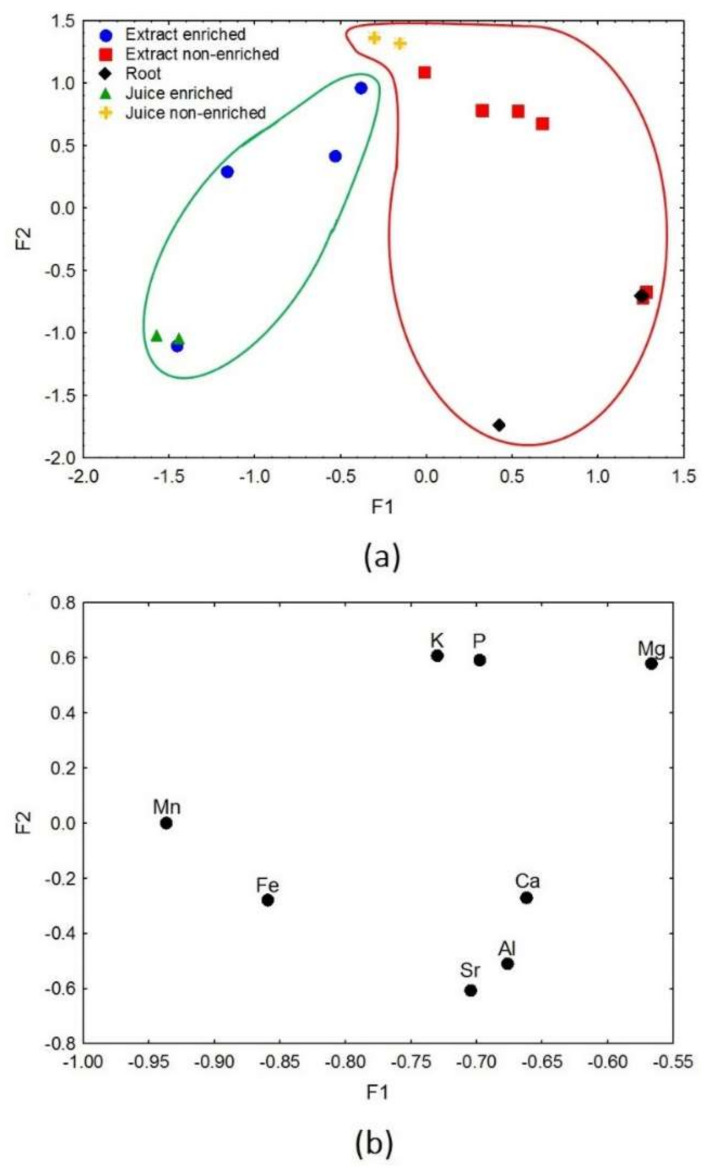
(**a**) Scatter plot of objects samples of two factors for the dietary supplements which were categorized accordingly: Fe-enriched (green line) and non-enriched (red line) products. (**b**) Scatter plot of loading for elements in all the analysed samples.

Factor analysis of dietary supplements dataset was performed using the following descriptors: K, P, Mg, Mn, Fe, Ca, Sr, and Al. The analysis resulted in the separation of dietary supplements’ samples in terms of various types of the main component (extract, root, and juice) as well as iron-enrichment (Figure 6a,b). The total value of the explained variance was 77.3%, of which F1 amounted to 54.4% while F2 to 22.9%. The eigenvalues for factors 1 and 2 were 4.35 and 1.83, respectively. Factor 1 was responsible for the diversification of dietary supplements’ samples according to the iron-enrichment of the product (Figure 6a). Higher values of F1, described by K, P, Mg, Ca, Sr, and Al, corresponded to samples of supplements containing root, non-enriched extracts, and juice. In turn, Mn and Fe were responsible for the distribution of iron-enriched dietary supplements (extracts and juice), which were described by lower F1 values. Higher values of F2 as well as K, P, and Mg corresponded mainly to the group of supplements’ samples with extracts and juice as their main components (Figure 6b). It might be supposed that the supplement manufacturer used the expressions extract-juice interchangeably in the declaration on the packaging. It was found that dietary supplements contained in their composition beetroot juice provided higher amounts of various elements, i.e., Fe, Mn, P, K, and Mg. On the other hand, the supplements made of root had a higher content of Ca, Al, and Sr. This analysis showed the importance of a dietary supplement’s choice according to the degree of processing of the main ingredient. It was noted that F2 also differentiated the analysed dietary supplement samples. However, due to the limited amount of information about products, it was not possible to demonstrate which feature is responsible for such distinction.

#### 3.5.5. Cluster Analysis

**Figure 7:**
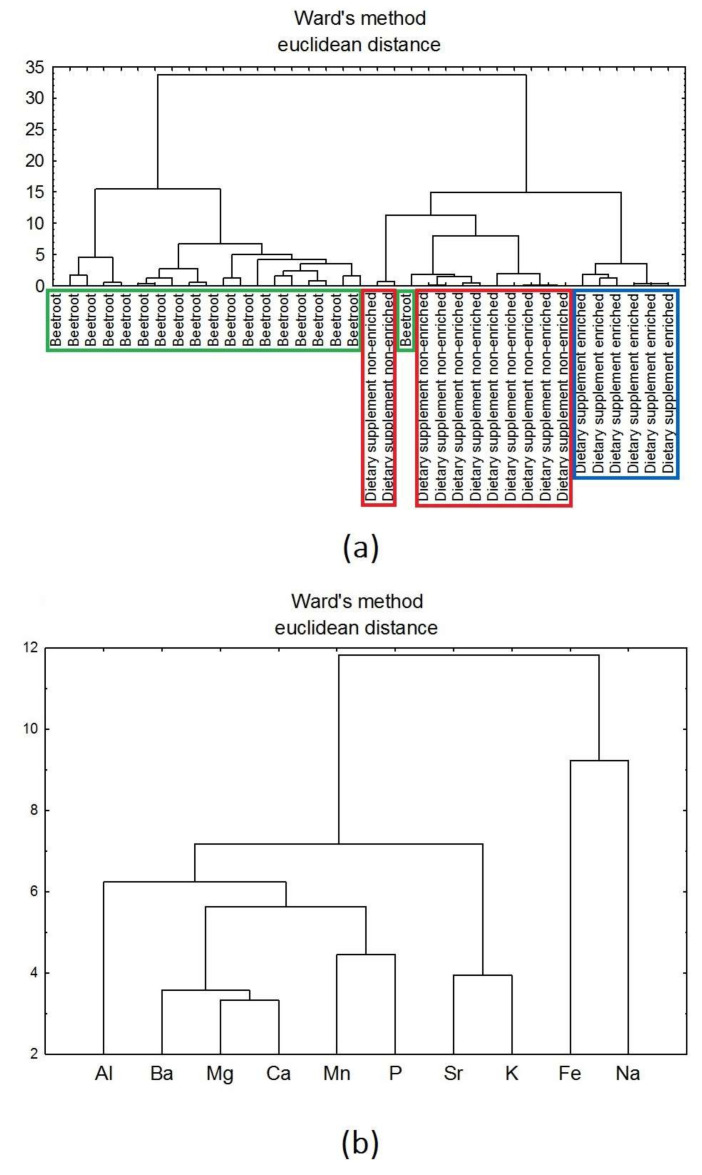
(**a**,**b**). Hierarchical dendrogram for all products (beetroot and Fe-enriched/non-enriched dietary supplements).

**Figure 8:**
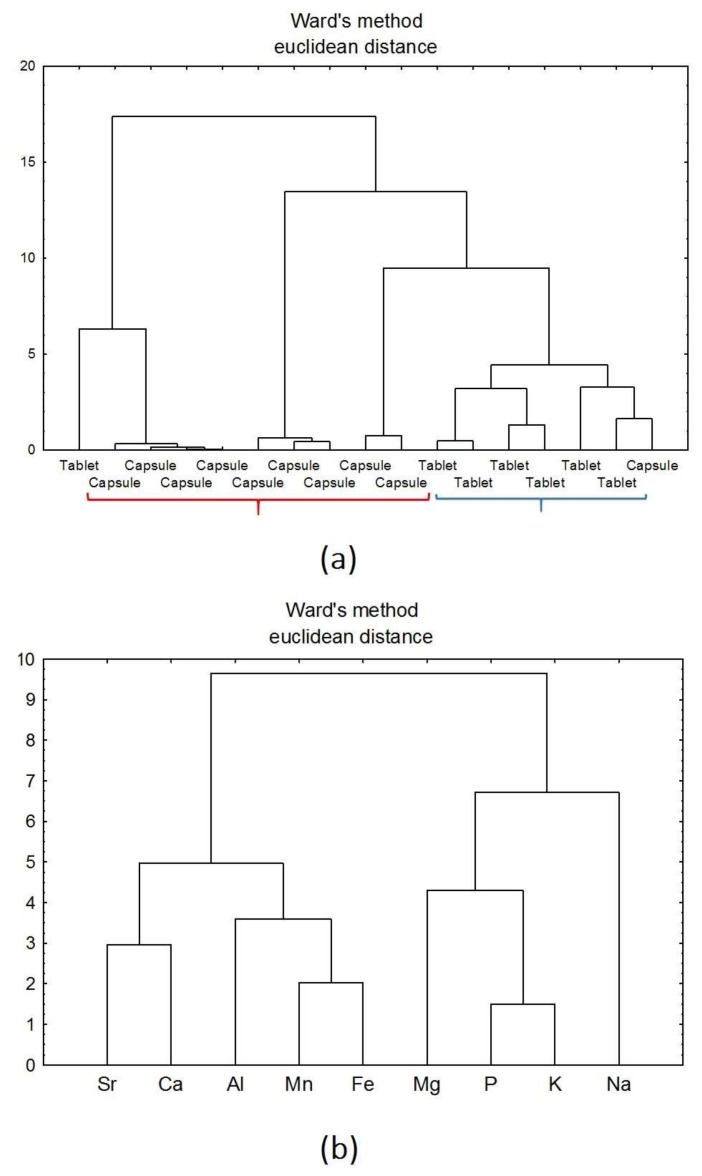
(**a**,**b**). Hierarchical dendrogram for the forms of dietary supplements (tablets and capsules).

Cluster analysis was performed using Ward’s method and the Euclidean distance. The study was performed for the datasets of all the analysed products (Figure 7a,b) and dietary supplements with a varied pharmaceutical form (Figure 8a,b).

The dendrogram (Figure 7) was built of two main clusters, one of which was assigned to the beetroot samples and the other to the dietary supplements. Beetroot samples were discriminated by Al, Ba, Mg, Ca, Mn, P, Sr, and K, and dietary supplements’ ones by Fe and Na (Figure 7b).

Application of CA allowed on differentiation of supplements samples according to their pharmaceutical form (tablet and capsule) (Figure 8). The dendrogram was built of two main clusters. There can be distinguished clusters concerning capsule and tablet dietary supplements’ samples (Figure 8a,b). The elements Sr, Ca, Al, Mn, and Fe were responsible for the distribution of products in the form of capsules. In the case of tablets, samples were discriminated by Mg, P, K, and Na. It was found that Na shows a strong dependence in the studied group of samples. One capsule, which was improperly assigned to the tablets cluster contained high levels of Na. Na might be a component of excipients used in the production of dietary supplements and this affected such diversification (Figure 8a). It was also found that one tablet was improperly assigned to the capsules clusters. This is probably due to poorly defined information on the tablet’s label by the manufacturer.

## 4. Conclusions

In this work, the mineral composition of beetroot-based dietary supplements and vegetables was successfully assessed and compared. In several cases, the supplements contained very small amounts of beetroot preserves, even less than 5 g expressed as a fresh vegetable. As a result, negligible amounts of micro- and macrominerals are provided with a daily portion of supplements in comparison with 100 g of beetroot. However, iron-enriched products were found to fulfil RDA for Fe significantly. Furthermore, some products were significantly contaminated with toxic elements (As, Cd), which might be associated with the accumulative abilities of the beetroot or contamination during production. Exposure to these products over an extended period could pose a significant health risk to consumers due to the poisoning with these elements. Multivariate techniques allowed the differentiation of beetroot and its supplements in view of their type, origin, type of cultivation, and form. Moreover, the factor analysis resulted in differentiation of organic and conventional beetroot samples based on their mineral composition. Chemometric techniques proved to be helpful in the verification of the authenticity and safety of the analysed products.

In conclusion, the analysed dietary supplements contained lower amounts of micro- and macrominerals than beetroot. Moreover, the safety of the final product should be assessed before releasing it to the market. Possible contamination with elements above the permissible limits or adulteration can be associated with a direct threat to the consumers’ health. Therefore, more stringent control of the dietary supplement market is necessary to provide consumer safety.

## Figures and Tables

**Table 1:** Full characteristics of the analysed beetroot samples based on information in the place of purchase or label.

Form	Code	Water Content (%)	Date of Purchase	Certificate of Organic Cultivation	Place of Purchase	Origin Country
Conventional	1Bo	85.9	11/05/2019	conventional cultivation	large-area shop, Gdańsk (PL)	Poland (PL)
	3Bo	85.2	11/14/2019	conventional cultivation	retail shop, Kolbudy (PL)	Poland (PL)
	4Bo	81.8	11/14/2019	conventional cultivation	large-area shop, Gdańsk (PL)	Poland (PL)
	5Bo	88.1	11/28/2019	conventional cultivation	large-area shop, Gdańsk (PL)	Poland (PL)
Organic	2Bo	83.2	11/05/2019	P 095 18, region: Greater Poland (PL)	large-area shop, Gdańsk (PL)	Poland (PL)
	6Bo	85.2	12/02/2019	PL-EKO-07-07904 Wilkowa Wieś, region: Pomeranian (PL)	grocery store (Internet), Gdańsk (PL)	Poland (PL)
	7Bo	83.4	12/02/2019	PL-EKO-07-07904 Wilkowa Wieś, region: Pomeranian (PL)	grocery store (Internet), Gdańsk (PL)	Poland (PL)

Bo–peeled beetroot.

**Table 2:** Full characteristics of the analysed beetroot-based dietary supplements according to information on the package (* iron-enriched products).

Form	Code	Number of Dosage Units	Product Net Weight (g)	The Content of Beetroot Extract or Preserves/Dosage Unit	Declared Weight of the Dosage Unit (g)	Recommendation (Dosage Units/Day)	Origin Country
capsules	1GyA	90	45	400 mg of root extract; 40 mg of nitrates	0.5	1 × 1 caps.	Poland (PL)
	1GyB						
	2PhA	90	45	400 mg of root extract (15:1); gelatine	0.5	1 × 1 caps.	Poland (PL)
	2PhB						
	3GaA *	60	35.76	dried juice concentrate; 38 mg of vitamin C; 2.8 mg of iron; capsule shell (gelatine of animal origin)	0.596	2 × 1 caps. during meal	Poland (PL)
	3GaB *						
	4HeA *	30	11.3	268 mg of beetroot concentrate; 20 mg of vitamin C; 12 mg (1.4 mg iron) of iron gluconate; starch; anti-caking agent: magnesium salts of fatty acids; silicon dioxide	0.376	1 × 3 caps.	Poland (PL)
	4HeB *						
	5DoA	60	33	500 mg of dried juice concentrate (refers to 2.75 g fresh beetroot); 1 mg of B_6_; 1.25 μg of B_12_; bulking agent: microcrystalline cellulose; anti-caking agents: fatty magnesium salts, silicon dioxide	0.55	1—2 × 3 caps.	Poland (PL)
	5DoB						
	9SoA	60	41.4	550 mg of *Beta vulgaris* extract 4:1; pullulan capsule	0.69	1 × 2 caps.	Poland (PL)
	9SoB						
tablets	6HeA *	60	39	488 mg of beetroot concentrate; 20 mg of vitamin C; 12 mg (1.4 mg iron) of iron gluconate; starch; anti-caking agent: magnesium salts of fatty acids; silicon dioxide	0.65	1 × 3	Poland (PL)
	6HeB *						
	7CoA	120	111	500 mg of dried juice (refers to 3.5 g of fresh beetroot); anti-caking agent: magnesium salts of fatty acids; silicon dioxide	0.925	1—2 × 3 tabl. during a meal or after a meal	Poland (PL)
	7CoB						
	8Sw	60	86	100 mg of beetroot root powder; 125 mg of L-arginine alpha-ketoglutarate; 125 mg of L-citrulline; 100 mg of Beta alanine; sweeteners: mannitol, xylitol and steviol glycosides; bulking agent: microcrystalline cellulose; stabilizer: sodium carboxymethylcellulose, cellulose gum; capsule shell: hydroxypropylmethyl cellulose; acidity regulator: citric acid; natural flavours (cherry and vanilla); emulsifier: hydroxypropyl cellulose; anti-caking agents: calcium salts of fatty acids and silicon dioxide	1.42	1 × 1—2 tabl. 20–30 min before training	United States (USA)

**Table 3:** Validation parameters of the procedure for the determination of selected elements in samples of beetroot and beetroot-based dietary supplements.

Analyte	Wavelength (nm)	LOD (mg/kg)	LOQ (mg/kg)	Linearity						Recovery for Calibration Curves (*R*_cc_) (%)	Precision (Expressed as CV)
				Calibration Range (mg/kg)		Number of Measurement Points	Number of Repetitions	Calibration Curve	*R* ^2^		
				Minimum Concentration	Maximum Concentration						
Na	589.592	0.61	1.8	1.8	35	6	3	y = 1.6 × 10^5^x + 1.0 × 10^4^	0.9988	8.6	6.6
K	766.491	0.24	0.72	4.9	35	5	3	y = 4.9 × 10^4^x + 4.0 × 10^4^	0.9998	7.8	4.6
P	213.618	3.6	11	11	44	8	4	y = 1.2 × 10^2^x + 6.4 × 10^2^	0.9857	6.2	3.6
Fe	371.993	0.29	0.87	0.87	28	7	4	y = 5.9 × 10^3^x + 9.1 × 10^2^	0.9950	8.8	8.1
Ca	393.366	0.067	0.20	0.49	17	5	4	y = 3.1 × 10^5^x + 3.1 × 10^4^	0.9958	8.1	4.4
As	197.198	0.10	0.30	2.9	11	4	4	y = 1.4 × 10^2^x + 1.3 × 10^2^	0.9955	4.8	1.8
Se	203.985	0.10	0.30	0.30	11	6	4	y = 2.3 × 10^2^x + 2.1 × 10^2^	0.9984	4.3	2.6
Zn	213.857	0.32	0.96	0.96	33	5	4	y = −9.1 × 10^2^x^2^ + 7.2 × 10^3^x + 9.3 × 10^2^	0.9994	7.2	4.6
Cd	228.802	0.23	0.69	0.69	11	6	4	y = 1.1 × 10^4^x − 9.8 × 10^2^	0.9991	15	3.5
Mg	383.829	0.11	0.33	0.49	100	10	4	y = 2.6 × 10^3^x + 1.6 × 10^3^	0.9972	6.0	2.4
Pb	368.346	0.057	0.17	0.17	11	6	4	y = 1.7 × 10^3^x − 2.0 × 10^2^	0.9998	14	6.6
Cu	324.754	0.070	0.21	0.21	11	5	4	y = 8.4 × 10^4^x + 8.4 × 10^3^	0.9994	16	6.0
Ag	328.068	0.28	0.84	4.9	11	4	4	y = 3.9 × 10^2^x + 9.8 × 10^2^	0.9992	1.8	8.9
Co	340.512	0.030	0.090	0.090	11	4	4	y = 6.2 × 10^3^x + 1.7 × 10^3^	0.9939	9.3	5.6
Ni	341.476	0.15	0.45	2.9	11	4	4	y = 1.3 × 104x + 2.4 × 10^3^	0.9997	2.2	1.2
Mo	379.825	0.053	0.16	0.16	11	4	4	y = 2.1 × 10^4^x − 1.5 × 10^3^	0.9997	5.9	2.9
Al	396.152	0.27	0.81	2.9	33	7	4	y = 2.1 × 10^4^x − 1.0 × 10^4^	0.9987	4.7	2.0
Mn	403.076	0.052	0.16	0.16	33	8	4	y = 2.4 × 10^4^x + 2.5 × 10^3^	0.9987	6.2	3.1
Sr	407.771	0.066	0.20	0.20	11	6	4	y = 1.6 × 10^5^x + 2.5 × 10^4^	0.9993	9.9	8.1
Cr	425.433	0.021	0.063	0.063	5	5	4	y = 2.6 × 10^4^x − 2.8 × 10^2^	0.9998	14	4.7
Ba	614.171	0.10	0.30	0.30	11	5	4	y = 6.2 × 10^4^x+1.7 × 10^3^	0.9974	7.9	6.7
Li	670.784	0.045	0.14	1	5	4	4	y = 1.0 × 10^6^x − 2.1 × 10^5^	0.9999	0.89	3.5

**Table 4:** Macro- and microelements’ contents in beetroot samples and realisation of dietary recommendations for adult male from 19 to 75 years old by a 100 g portion of the analysed beetroots.

Analysed Element	Dietary Recommendations (mg/day)	Beetroot Samples													
		Conventional							Organic						
		*n* ^1^	(mg/100 g f.w.)					Realisation of Dietary Recommendations (%)	*n* ^1^	(mg/100 g f.w.)					Realisation of Dietary Recommendations (%)
			Mean	SD	Min	Median	Max			Mean	SD	Min	Median	Max	
Na	1500 ^a^	12	35	16	19	36	52	2.4 ^a^	9	32	25	17	19	61	2.1 ^a^
K	3500 ^a^	12	266	37	215	277	295	7.6 ^a^	9	356	149	261	279	527	10 ^a^
P	700 ^b^	12	20.8	5.3	16	19	28	3.0 ^b^	9	37.54	0.56	37	38	38	5.4 ^b^
Mg	420 ^b^	12	22.4	4.7	16	24	27	5.3 ^b^	9	30.2	7.4	24	29	38	7.2 ^b^
Ca	1000 ^b^	12	21.7	3.2	18	22	26	2.2 ^b^	9	34	14	25	27	51	3.4 ^b^
Fe	10 ^b^	12	0.68	0.14	0.50	0.70	0.83	6.8 ^b^	9	0.82	0.11	0.70	0.88	0.88	8.2 ^b^
Se	0.055 ^b^	3	0.541	0.032	0.51	0.54	0.58	245 ^b^	9	<LOQ					<LOQ
Zn	11 ^b^	6	0.380	0.039	0.35	0.38	0.41	1.7 ^b^	9	<LOQ					<LOQ
Cu	0.9 ^b^	9	0.097	0.013	0.082	0.10	0.11	8.1 ^b^	9	<LOQ					<LOQ
Mn	2.3 ^a^	12	0.58	0.71	0.17	0.25	1.6	25 ^a^	9	0.36	0.10	0.25	0.41	0.42	16 ^a^
Sr	NR	12	0.138	0.030	0.11	0.13	0.18	NR	9	0.24	0.23	0.093	0.12	0.50	NR
Ba	NR	12	0.175	0.054	0.12	0.16	0.25	NR	9	0.217	0.022	0.19	0.22	0.24	NR
Al	NR	9	0.65	0.18	0.46	0.66	0.82	NR	9	0.85	0.92	0.23	0.4	1.9	NR
As	NR	3	3.246	0.047	3.21	3.23	3.30	NR	3	3.68	0.11	3.56	3.67	3.78	NR
Cd	NR	3	0.06387	0.00013	0.0638	0.0638	0.0640	NR	9	<LOQ					<LOQ

SD—standard deviation, Min—minimum, Max—maximum, *n*^1^—number of samples with the determined content of analysed element above LOQ (LOQ Se = 0.30 µg/g, LOQ Zn = 0.96 µg/g, LOQ Cu = 0.21 µg/g, LOQ Cd = 0.69 µg/g); ^a^ AI for man, ^b^ RDA for man, NR–lack of dietary recommendation.

**Table 5:** Macro- and microelements’ contents in capsulated and tableted beetroot-based dietary supplements samples and realisation of dietary recommendations for adult male from 19 to 75 years old by a daily portion of the analysed dietary supplements.

Analysed Element	Dietary Recommendations (mg/day)	Beetroot-Based Dietary Supplements													
		Capsules							Tablets						
		*n* ^1^	(µg/d.u.)					Realisation of Dietary Recommendations (%)	*n* ^1^	(µg/d.u.)					Realisation of Dietary Recommendations (%)
			Mean	SD	Min	Median	Max			Mean	SD	Min	Median	Max	
Na	1500 ^a^	10	1625	2576	303	469	6947	0.22 ^a^	7	1108	1012	287	870	3276	0.30 ^a^
K	3500 ^a^	10	3510	2440	680	5055	5943	0.22 ^a^	7	5421	2677	2617	5358	9010	0.81 ^a^
P	700 ^b^	6	871	137	108	862	1024	0.30 ^b^	6	724	249	438	677	1922	0.55 ^b^
Mg	420 ^b^	10	410	416	108	210	1297	0.24 ^b^	7	806	749	267	364	1922	1.0 ^b^
Ca	1000 ^b^	10	330	154	189	253	544	0.068 ^b^	7	626	530	271	421	1171	0.25 ^b^
Fe	10 ^b^	10	1004	1312	18	112	2945	24 ^b^	7	457	736	14	39	1576	14 ^b^
Zn	11 ^b^	2	4.21	0.46	3.9	4.2	4.5	0.097 ^b^	0	<LOQ					<LOQ
Mn	2.3 ^a^	4	17.1	2.9	14	17	20	1.8 ^a^	6	10.9	5.8	4.1	12	19	2.3 ^a^
Sr	NR	3	3.00	0.26	2.7	3.0	3.2	NR	2	2.14	0.27	2.0	2.1	2.3	NR
Al	NR	9	50.4	1.2	3.8	11	190	NR	6	37	71	4.4	9.8	182	NR
As	NR	3	93.9	1.2	93	94	95	NR	0	<LOQ					NR
Cd	NR	3	22	34	2.5	2.8	61	NR	2	2.518	0.019	2.5	2.5	2.5	NR

SD—standard deviation, Min—minimum, Max—maximum, *n*^1^—number of samples with the determined content of analysed element above LOQ (LOQ Zn = 0.96 µg/g, LOQ As = 0.30 µg/g); d.u.—dosage unit, ^a^ AI for man, ^b^ RDA for man, NR–lack of dietary recommendation.

**Table 6:** Compliance of the determined iron content with manufacturers’ declarations and guidelines.

Sample	Declared Iron Content (mg/d.u.)	Accepted Minimum Tolerance (−20%)	Accepted Maximum Tolerance (+45%)	Determined Iron Content (mg/d.u.)	Compliance with the Declaration (%)	Compliance with the Guidelines
3GaA	2.8	2.24	4.06	2.80	100	YES
3GaB	2.8	2.24	4.06	2.72	97	YES
6HeA	1.4	1.12	2.03	1.28	91	YES
6HeB	1.4	1.12	2.03	2.95	210	NO
4HeA	1.4	1.12	2.03	1.49	107	YES
4HeB	1.4	1.12	2.03	1.58	113	YES

**Table 7:** Determined content of Cd in beetroot and dietary supplement samples expressed as a percentage of the maximum allowable level of its contamination.

Product	*n* ^1^	Mean (%)	SD (%)	Min (%)	Max (%)	Q1 (%)	Median (%)	Q3 (%)	Permissible Contamination Limit (mg/kg f.w.)
beetroot samples									0.06
conventional	3	1064.53	2.17	1063	1067	1063	1065	1066	
dietary supplements									1.0
capsules	3	4430	6757	504	12,233	529	554	6394	
tablets	2	503.7	3.8	501	506	502	504	505	

SD—standard deviation, Min—minimum, Max—maximum, Q1—lower quartile, Q3—upper quartile; *n*^1^—number of samples with the determined content of Cd above LOQ (All validation parameters-Table 3).

## Data Availability

All data are contained within the article and Supplementary Materials.

## Supplementary Materials

The following are available online at https://www.mdpi.com/article/10.3390/nu14010106/s1, Table S1: The average recovery for the most of the elements to be determined (* value obtained for information value). Table S2: Results of the determination of the selected elements’ content in conventional and organic beetroot samples (x_m_ ± U, (k = 2)). Table S3: Results of the determination of the content of selected elements in beetroot-based food supplements samples (x_m_ ± U, (k = 2)). Table S4: Results of the realisation of dietary recommendation (%) for selected elements by a 100 g portion of conventional and organic beetroot samples. Table S5: Results of the realisation of dietary recommendation (%) for selected elements by a daily portion of dietary supplements. Table S6: Spearman’s rank correlation of beetroot and dietary supplements samples (red font for statistically significant correlations). Table S7: Spearman’s rank correlation of beetroot samples (red font for statistically significant correlations). Table S8: Spearman’s rank correlation of dietary supplements samples (red font for statistically significant correlations). Table S9: Relationships between the category of the analysed samples and the concentration of elements. Table S10: Results of the Dunn’s test for all the analysed samples (beetroot and dietary supplements).
