# Supplementary material for: Mineral Composition of Dietary Supplements-Analytical and Chemometric Approach
Source: Nutrients. 2021 Dec 27;14(1):106. doi: 10.3390/nu14010106 (PMC8746997; doi:10.3390/nu14010106)
Supplement: Supplementary file 1 [file nutrients-14-00106-s001.zip › nutrients-1484057-supplementary.pdf]

Table S1. The average recovery for the most of the elements to be determined (*value obtained for information value).

Analyte	Recovery (%)			
	**M–4 CormTis**	**M–3 HerTis**	**M–5 CodTis**	**DOLT-4**
Na	101.8 ± 4.9	99.8 ± 9.6	107 ± 12	-
K	89.2 ± 2.1	105 ± 11	105 ± 11	-
P	84.5 ± 3.9	86.2 ± 4.1	87.6 ± 6.0	-
Fe	112.9 ± 4.8	104 ± 10	97.5 ± 6.1	94.6 ± 9.7
Ca	99.7 ± 8.1*	-	-	-
As	89.7 ± 6.3	85.1 ± 4.0	87.9 ± 6.3	85.2 ± 4.9
Se	83.9 ± 1.4	96.8 ± 9.7	90.4 ± 4.9	86.2 ± 5.4
Zn	102.1 ± 5.6	93.5 ± 7.4	110.1 ± 6.9	96.3 ± 3.7
Cd	94.0 ± 7.8	90.2 ± 3.9	-	87.8 ± 5.9
Mg	98.4 ± 5.7	107.9 ± 6.1	97.5 ± 8.4	-
Pb	94.4 ± 6.1	88.7 ± 6.5	-	92.0 ± 7.3
Cu	94.8 ± 8.1	93.1 ± 8.1	86.2 ± 5.1	91.2 ± 6.4
Ag	-	87.6 ± 3.8	-	-
Co	95.7 ± 7.1	87.0 ± 5.1	-	-
Ni	-	84.9 ± 3.7	-	86.3 ± 4.7
Mo	-	85.0 ± 3.0	-	-
Al	-	-	-	-
Mn	88.9 ± 5.3	98.0 ± 2.7	95.2 ± 5.9	-
Sr	90.7 ± 7.0	-	88.7 ± 7.0	-
Cr	-	100.1 ± 6.9	-	93.7 ± 6.3
Ba	-	87.6 ± 5.0	90.1 ± 8.4	-
Li	-	88.6 ± 3.4	-	-

Table S2. Results of the determination of the selected elements' content in conventional and organic beetroot samples (x_m ± U, (k = 2)).

Type	No.	Sample	Concentration ($Cont_1$) ± U (mg/100 g f.w.)														
			**Na**	**K**	**P**	**Mg**	**Ca**	**Fe**	**Se**	**Zn**	**Cu**	**Mn**	**Sr**	**Ba**	**Al**	**As**	**Cd**
conventional	1.	1Bo	51.64 ±0.53	263.32 ±0.79	28.48 ±0.18	24.47 ±0.20	25.54 ±0.41	0.6971 ±0.0039	<LOQ	<LOQ	0.08166 ±0.00080	1.640 ±0.013	0.10518 ±0.00056	0.15049 ±0.00062	0.4588 ±0.0018	3.246 ±0.090	<LOQ
	2.	3Bo	18.539 ±0.050	214.63 ±0.94	19.19 ±0.21	15.84 ±0.13	22.23 ±0.97	0.7124 ±0.0031	0.541 ±0.064	0.4075 ±0.0043	0.1058 ±0.0010	0.174 ±0.016	0.1782 ±0.0010	0.2483 ±0.0016	0.6573 ±0.0092	<LOQ	<LOQ
	3.	4Bo	46.28 ±0.15	291.5 ±1.94	16.37 ±0.33	26.64 ±0.22	21.68 ±0.18	0.8263 ±0.0042	<LOQ	<LOQ	<LOQ	0.250 ±0.0031	0.13330 ±0.00056	0.12181 ±0.00031	0.8190 ±0.0076	<LOQ	<LOQ
	4.	5Bo	24.84 ±0.91	294.63 ±0.57	19.12 ±0.52	22.82 ±0.12	17.55 ±0.40	0.50106 ±0.00042	<LOQ	0.353 ±0.0016	0.10370 ±0.00087	0.2433 ±0.0022	0.13402 ±0.00027	0.17946 ±0.00039	<LOQ	<LOQ	0.0639 ±0.0010
organic	5.	2Bo	61.04 ±0.57	527.3 ±1.36	38.09 ±0.10	38.31 ±0.20	51.05 ±0.41	0.883 ±0.055	<LOQ	<LOQ	<LOQ	0.4227 ±0.0041	0.5003 ±0.0030	0.22311 ±0.00073	1.905 ±0.020	3.684 ±0.095	<LOQ
	6.	6Bo	16.54 ±0.10	260.6 ±1.54	36.97 ±0.30	23.73 ±0.15	25.38 ±0.43	0.698 ±0.071	<LOQ	<LOQ	<LOQ	0.2460 ±0.0026	0.0934 ±0.0026	0.19275 ±0.0016	0.2265 ±0.0035	<LOQ	<LOQ
	7.	7Bo	19.05 ±0.22	279.3 ±1.0	37.56 ±0.22	28.64 ±0.18	26.82 ±0.15	0.8766 ±0.0028	<LOQ	<LOQ	<LOQ	0.4062 ±0.0022	0.1153 ±0.0030	0.23533 ±0.00092	0.4066 ±0.0094	<LOQ	<LOQ

LOQ Se=0.30 μg/g, LOQ Zn=0.96 μg/g, LOQ Cu=0.21 μg/g, LOQ Al=0.81 μg/g, LOQ As=0.30 μg/g, LOQ Cd=0.69 μg/g; U – expanded uncertainty of measurement at 95% confidence level obtained for three replicates

Table S3. Results of the determination of the content of selected elements in beetroot-based food supplements samples (x_m ± U, (k = 2)).

Form	No.	Sample	Concentration ± U (µg/d.u. of product)											
			Na	K	P	Mg	Ca	Fe	Zn	Mn	Sr	Al	As	Cd
capsules	1.	1GyA	302.94 ±4.35	679.50 ±4.35	<LOQ	107.64 ±0.45	218.15 ±0.60	19.91 ±0.032	<LOQ	<LOQ	<LOQ	8.75 ±0.28	<LOQ	61.17 ±0.46
	2.	1GyB	328.55 ±3.75	698.0 ±0.024	<LOQ	112.17 ±0.33	192.90 ±1.85	18.75 ±0.022	<LOQ	<LOQ	<LOQ	17.38 ±0.25	93.90 ±3.40	<LOQ
	3.	2PhA	323.65 ±4.35	690.950 ±0.024	<LOQ	109.10 ±0.35	192.60 ±2.10	18.48 ±0.075	<LOQ	<LOQ	<LOQ	20.61 ±0.25	95.0 ±1.10	<LOQ
	4.	2PhB	317.0 ±5.50	700.950 ±0.024	<LOQ	111.10 ±0.40	188.70 ±1.70	18.32 ±0.15	<LOQ	<LOQ	<LOQ	11.31 ±0.14	92.70 ±2.15	<LOQ
	5.	3GaA	474.36 ±2.26	5409.296 ±0.029	660 ±15	488.06 ±2.21	488.72 ±1.55	2797.0 ±2.32	3.880 ±0.089	19.751 ±0.066	2.724 ±0.024	221.65 ±2.92	<LOQ	3.302 ±0.032
	6.	3GaB	477.63 ±2.03	5206.060 ±0.029	862.41 ±5.96	487.23 ±2.26	520.61 ±2.26	2720 ±12	<LOQ	19.38 ±0.15	3.041 ±0.027	226.54 ±0.72	<LOQ	<LOQ
	7.	6HeA	464.36 ±4.42	4948.450 ±0.031	952.97 ±1.17	971.43 ±5.59	464.82 ±2.99	1275.30 ±7.80	<LOQ	14.869 ±0.051	<LOQ	6.58 ±0.25	<LOQ	<LOQ
	8.	6HeB	577.20 ±6.50	5943.080 ±0.031	855 ±21	1297.01 ±2.60	543.99 ±2.21	2945 ±18	4.53 ±0.12	14.235 ±0.072	3.239 ±0.027	<LOQ	<LOQ	3.278 ±0.042
	9.	9SoA	6040 ±37	5161.890 ±0.033	<LOQ	194.85 ±0.69	203.76 ±1.24	203.78 ±0.51	<LOQ	<LOQ	<LOQ	5.24 ±0.15	<LOQ	<LOQ
	10.	9SoB	6947 ±10	5663.520 ±0.033	1023.96 ±8.28	225.35 ±1.52	287.52 ±0.61	18.22 ±0.17	<LOQ	<LOQ	<LOQ	14.80 ±0.083	<LOQ	<LOQ
tablets	11.	4HeA	287.26 ±6.39	2999.728 ±0.018	512 ±11	266.51 ±1.65	270.83 ±1.17	1492 ±23	<LOQ	11.22 ±0.11	<LOQ	3.83 ±0.23	<LOQ	<LOQ
	12.	4HeB	336.90 ±3.76	3321.584 ±0.018	438.45 ±1.28	311.29 ±0.79	324.49 ±5.26	1576 ±18	<LOQ	12.34 ±0.094	1.9514 ±0.0064	136.90 ±0.22	<LOQ	1.9037 ±0.0094
	13.	5BoA	774.95 ±6.60	5358.100 ±0.027	631.95 ±9.90	306.50 ±0.30	290.35 ±0.99	14.413 ±0.030	<LOQ	4.058 ±0.014	<LOQ	10.58 ±0.46	<LOQ	<LOQ
	14.	5DoB	870 ±10	5777.20 ±0.027	722.70 ±8.80	616.28 ±4.07	421.262 ±0.050	15.752 ±0.044	<LOQ	4.472 ±0.048	<LOQ	4.862 ±0.019	<LOQ	<LOQ
	15.	7CoA	1057 ±16	9009.50 ±0.045	1056 ±10	1922 ±13	687.83 ±3.89	41.656 ±0.027	<LOQ	18.65 ±0.18	<LOQ	18.32 ±1.20	<LOQ	<LOQ
	16.	7CoB	1155.14 ±4.16	8865.20 ±0.045	979.95 ±8.70	1857 ±13	611.61 ±3.61	39.36 ±0.17	<LOQ	14.91 ±0.10	<LOQ	21.47 ±0.29	<LOQ	<LOQ
	17.	8SwA	3275.94 ±4.12	2617.060 ±0.069	<LOQ	363.52 ±1.99	1771 ±21	20.79 ±0.26	<LOQ	<LOQ	2.330 ±0.037	<LOQ	<LOQ	7.11 ±0.16

LOQ P=11 µg/g, LOQ Zn=0.96 µg/g, LOQ Mn=0.16 µg/g, LOQ Sr=0.20 µg/g, LOQ Al=0.81 µg/g, LOQ As=0.30 µg/g, LOQ Cd=0.69 µg/g; U – expanded uncertainty of measurement at 95% confidence level obtained for three replicates

Table S4. Results of the realisation of dietary recommendation (%) for selected elements by a 100 g portion of conventional and organic beetroot samples.

Type	No.	Sample	Realisation of Dietary Recommendation (%)									
			Na	K	P	Mg	Ca	Fe	Se	Zn	Cu	Mn
			AI 1500 mg/day	AI 3500 mg/day	RDA 700 mg/day	RDA men 420 mg/day	RDA 1000 mg/day	RDA men 10 mg/day	RDA 0.055 mg/day	RDA men 11 mg/day	RDA 0.9 mg/day	AI men 2.3 mg/day
conventional	1.	1Bo	3.44	7.52	4.07	5.83	2.55	6.97	<LOQ	<LOQ	9.07	91.1
	2.	3Bo	1.24	6.13	2.74	3.77	2.22	7.12	983	3.7	11.8	9.69
	3.	4Bo	3.09	8.33	2.34	6.34	2.17	8.26	<LOQ	<LOQ	<LOQ	13.9
	4.	5Bo	1.66	8.42	2.73	5.43	1.75	5.01	<LOQ	3.21	11.5	13.5
organic	5.	2Bo	4.07	15.1	5.44	9.12	5.10	8.83	<LOQ	<LOQ	<LOQ	18.4
	6.	6Bo	1.10	7.44	5.28	5.65	2.54	6.98	<LOQ	<LOQ	<LOQ	10.7
	7.	7Bo	1.27	7.98	5.37	6.82	2.68	8.77	<LOQ	<LOQ	<LOQ	17.7

LOQ P=11 μg/g, LOQ Se=0.30 μg/g, LOQ Zn=0.96 μg/g, LOQ Cu=0.21 μg/g; AI and RDA values according to Jarosz et al. [27]

Table S5. Results of the realisation of dietary recommendation (%) for selected elements by a daily portion of dietary supplements.

Form	No.	Sample	Realisation of Dietary Recommendation (%)							
			Na<br>AI<br>1500 mg/day	K<br>AI<br>3500 mg/day	P<br>RDA<br>700 mg/day	Mg<br>RDA men<br>420 mg/day	Ca<br>RDA<br>1000 mg/day	Fe<br>RDA men<br>10 mg/day	Zn<br>RDA men<br>11 mg/day	Mn<br>AI men<br>2.3 mg/day
capsules	1.	1GyA	0.02	0.02	<LOQ	0.03	0.02	0.20	<LOQ	<LOQ
	2.	1GyB	0.02	0.02	<LOQ	0.03	0.02	0.19	<LOQ	<LOQ
	3.	2PhA	0.02	0.02	<LOQ	0.03	0.02	0.18	<LOQ	<LOQ
	4.	2PhB	0.02	0.02	<LOQ	0.03	0.02	0.18	<LOQ	<LOQ
	5.	3GaA	0.06	0.31	0.19	0.23	0.10	55.9	0.07	1.72
	6.	3GaB	0.06	0.30	0.25	0.23	0.10	54.4	<LOQ	1.69
	7.	6HeA	0.09	0.42	0.41	0.69	0.14	38.3	<LOQ	1.94
	8.	6HeB	0.12	0.51	0.37	0.93	0.16	88.4	0.12	1.86
	9.	9SoA	0.81	0.29	<LOQ	0.09	0.04	4.08	<LOQ	<LOQ
	10.	9SoB	0.93	0.32	0.29	0.11	0.06	0.36	<LOQ	<LOQ
tablets	11.	4HeA	0.06	0.26	0.22	0.19	0.08	44.7	<LOQ	1.46
	12.	4HeB	0.07	0.28	0.19	0.22	0.10	47.3	<LOQ	1.61
	13.	5BoA	0.31	0.92	0.54	0.44	0.17	0.86	<LOQ	1.06
	14.	5DoB	0.35	0.99	0.62	0.88	0.25	0.95	<LOQ	1.17
	15.	7CoA	0.42	1.54	0.91	2.75	0.41	2.50	<LOQ	4.86
	16.	7CoB	0.46	1.52	0.84	2.65	0.37	2.36	<LOQ	3.89
	17.	8SwA	0.44	0.15	<LOQ	0.17	0.35	0.42	<LOQ	<LOQ

LOQ P=11 μg/g, LOQ Zn=0.96 μg/g, LOQ Mn=0.16 μg/g; AI and RDA values according to Jarosz et al. [27]

Table S6. Spearman's rank correlation of beetroot and dietary supplements samples (red font for statistically significant correlations).

	Na	K	P	Fe	Ca	Mg	Al	Mn	Sr	Ba
Na	1.000000	0.604376[abc]	0.457918[ab]	-0.059202	0.516345[ab]	0.459717[ab]	0.053445	0.259625	0.479073[ab]	0.396698[a]
K	0.604376[abc]	1.000000	0.679524[abc]	0.159588	0.867954[abc]	0.811326[abc]	0.250097	0.587449[abc]	0.858606[abc]	0.787954[abc]
P	0.457918[ab]	0.679524[abc]	1.000000	0.396260[a]	0.646503[abc]	0.654371[abc]	0.179348	0.618034[abc]	0.565329[abc]	0.594346[abc]
Fe	-0.059202	0.159588	0.396260[ab]	1.000000	0.340026[a]	0.458430[ab]	0.380554[a]	0.719974[abc]	0.249831	0.217395
Ca	0.516345[ab]	0.867954[abc]	0.646503[abc]	0.340026[ab]	1.000000	0.831918[abc]	0.429105[ab]	0.722557[abc]	0.895982[abc]	0.856791[abc]
Mg	0.459717[ab]	0.811326[abc]	0.654371[abc]	0.458430[ab]	0.831918[abc]	1.000000	0.334321[a]	0.758207[abc]	0.697167[abc]	0.787137[abc]
Al	0.053445	0.250097	0.179348	0.380554[a]	0.429105[ab]	0.334321[a]	1.000000	0.465686[ab]	0.396772[a]	0.406891[a]
Mn	0.259625	0.587449[abc]	0.618034[abc]	0.719974[abc]	0.722557[abc]	0.758207[abc]	0.465686[ab]	1.000000	0.535431[abc]	0.575084[abc]
Sr	0.479073[ab]	0.858606[abc]	0.565329[abc]	0.249831	0.895982[abc]	0.697167[abc]	0.396772[a]	0.535431[abc]	1.000000	0.836054[abc]
Ba	0.396698[a]	0.787954[abc]	0.594346[abc]	0.217395	0.856791[abc]	0.787137[abc]	0.406891[a]	0.575084[abc]	0.836054[abc]	1.000000

a =$p < 0.05$; b = $p < 0.01$; c = $p < 0.001$

Table S7. Spearman's rank correlation of beetroot samples (red font for statistically significant correlations).

	Na	K	P	Fe	Ca	Mg	Al	Mn	Sr	Ba
Na	1.000000	0.670175[ab]	0.238596	0.366667	0.642105[ab]	0.449123	0.328214	0.563158[a]	0.464239[a]	-0.045614
K	0.670175[ab]	1.000000	0.278947	0.222807	0.526316[a]	0.461404[a]	0.021062	0.319298	0.587977[ab]	0.010526
P	0.238596	0.278947	1.000000	0.521053[a]	0.400000	0.512281[a]	0.188679	0.540351[a]	0.021939	0.345614
Fe	0.366667	0.222807	0.521053[a]	1.000000	0.812281[abc]	0.891228[abc]	0.719614[abc]	0.696491[abc]	0.273804	0.743860[abc]
Ca	0.642105[ab]	0.526316[a]	0.400000	0.812281[abc]	1.000000	0.812281[abc]	0.579201[ab]	0.689474[ab]	0.452830	0.440351
Mg	0.449123	0.461404[a]	0.512281[a]	0.891228[abc]	0.812281[abc]	1.000000	0.564283[a]	0.780702[abc]	0.300132	0.729825[abc]
Al	0.328214	0.021062	0.188679	0.719614[abc]	0.579201[ab]	0.564283[a]	1.000000	0.426503	0.214662	0.481790[a]
Mn	0.563158[a]	0.319298	0.540351[a]	0.696491[abc]	0.689474[ab]	0.780702[abc]	0.426503	1.000000	0.004388	0.457895[a]
Sr	0.464239[a]	0.587977[ab]	0.021939	0.273804	0.452830	0.300132	0.214662	0.004388	1.000000	0.190434
Ba	-0.045614	0.010526	0.345614	0.743860[abc]	0.440351	0.729825[abc]	0.481790[a]	0.457895[a]	0.190434	1.000000

a= $p < 0.05$; b= $p < 0.01$; c = $p < 0.001$

Table S8. Spearman's rank correlation of dietary supplements samples (red font for statistically significant correlations).

	Na	K	P	Fe	Ca	Mg	Al	Mn	Sr
Na	1.000000	0.556373[a]	0.414424	-0.272059	0.220588	0.350490	-0.338443	-0.045714	0.100360
K	0.556373[a]	1.000000	0.392090	0.144608	0.465686	0.816176[abc]	-0.036787	0.553649[a]	0.164226
P	0.414424	0.392090	1.000000	0.364792	0.201008	0.469019	-0.172576	0.447421	0.107771
Fe	-0.272059	0.144608	0.364792	1.000000	0.365196	0.453431	0.289393	0.787299[abc]	0.492677[a]
Ca	0.220588	0.465686	0.201008	0.365196	1.000000	0.536765[a]	-0.025751	0.675554[ab]	0.784633[abc]
Mg	0.350490	0.816176[abc]	0.469019	0.453431	0.536765[a]	1.000000	-0.068670	0.711109[ab]	0.228091
Al	-0.338443	-0.036787	-0.172576	0.289393	-0.025751	-0.068670	1.000000	0.289701	0.196279
Mn	-0.045714	0.553649[a]	0.447421	0.787299[abc]	0.675554[ab]	0.711109[ab]	0.289701	1.000000	0.537291[a]
Sr	0.100360	0.164226	0.107771	0.492677[a]	0.784633[abc]	0.228091	0.196279	0.537291[a]	1.000000

a= $p < 0.05$; b= $p < 0.01$; c = $p < 0.001$

Table S9. Relationships between the category of the analysed samples and the concentration of elements.

Category	Na	K	P	Fe	Ca	Mg	Al	Mn	Sr	Ba
Form of the product (vegetable-beetroot and dietary supplement)	9.767[b]	25.968[c]	14.278[c]	21.392[c]	27.056[c]	18.034[c]	4.690	19.052[c]	28.157[c]	29.262[c]
Origin of beetroot	17.765[a]	30.652[c]	20.193[b]	23.699[b]	28.856[c]	25.698[c]	11.337	24.358[b]	32.327[c]	30.927[c]
Type of main component of dietary supplement	2.451	7.010	7.584	12.647[a]	7.569	11.758[a]	5.804	14.368[b]	7.894[a]	-

$p < 0.05$ [a]; $p < 0.01$ [b]; $p < 0.001$ [c]

Table S10. Results of the Dunn's test for all the analysed samples (beetroot and dietary supplements).

	Beetroot	Dietary supplement enriched	Dietary supplement non-enriched
Beetroot	-	Na[b], K[b], Fe[a], Ca[a], Sr[b], Ba[c]	K[c], P[c], Fe[a], Ca[c], Mg[c], Mn[c], Sr[c], Ba[c]
Dietary supplement enriched	Na[b], K[b], Fe[a], Ca[a], Sr[b], Ba[c]	-	Fe[c], Mn[b]
Dietary supplement non-enriched	K[c], P[c], Fe[a], Ca[c], Mg[c], Mn[c], Sr[c], Ba[c]	Fe[c], Mn[b]	-

$p < 0.05$ [a]; $p < 0.01$ [b]; $p < 0.001$ [c]
